# Association of mannose-binding lectin gene polymorphisms with the development of pulmonary tuberculosis in China

**DOI:** 10.1186/s12879-017-2310-3

**Published:** 2017-03-15

**Authors:** Yan-Ling Guo, Yang Liu, Wu-Juan Ban, Qi Sun, Guang-Li Shi

**Affiliations:** 1 Clinical laboratory, Beijing Chest Hospital, Capital Medical University, Beijing Tuberculosis and Thoracic Tumor Research Institute, No. 97 Machang, Tongzhou District, Beijing, 101149 People’s Republic of China; 2 Laboratory of Epidemiology, Beijing Chest Hospital, Capital Medical University, Beijing Tuberculosis and Thoracic Tumor Research Institute, Beijing, 101149 China; 3 Molecular biology laboratory, Beijing Chest Hospital, Capital Medical University, Beijing Tuberculosis and Thoracic Tumor Research Institute, Beijing, 101149 China

**Keywords:** Mannose-binding lectin (MBL), Polymorphism, Susceptibility, Tuberculosis

## Abstract

**Background:**

Mannose-binding lectin (MBL) is an important protein in the lectin pathway of the immune system. This study explores the association between MBL polymorphism and the susceptibility to tuberculosis (TB). The association between the MBL2 polymorphisms and serum MBL levels is also analyzed in the present study.

**Methods:**

A total of 112 inpatients with pulmonary TB and 120 healthy controls were recruited to participate in this case–control study. Polymerase Chain Reaction-Restriction Fragment Length Polymorphism(P**C**R-RFLP) technology was used to genotype MBL gene (variants in −221Y/X and exon l codons 54 A/B). Serum MBL level was assayed by human MBL ELISA kit. Demographic data and exposure information were also obtained from the study participants.

**Results:**

Genotypes YA/YA of MBL gene were more prevalent in the healthy control group than in the TB patient (*P* =0.038, OR, 0.57; 95% CI, 0.34-0.97) and genotypes XA/XA were less frequent in the healthy control group (*P* =0.007, OR, 6.42; 95% CI, 1.39-29.67). The resistant diplotype was more frequently found in the younger patients and retreatment cases with TB in MBL gene sites −221Y/X or codon 54 A/B. X/Y and A/B polymorphisms were strong determinants of serum MBL levels.

**Conclusion:**

The polymorphisms of MBL gene may be associated with susceptibility to TB and the recurrence of TB. The YA/YA may be a protected diplotype against TB.

## Background

Tuberculosis (TB), an infectious disease caused by *Mycobacterium tuberculosis (M. tuberculosis)*, is responsible for the mortality of approximately two million patients annually. TB is exacerbated by the emergence of multidrug-resistant and extensively drug-resistant (MDR and XDR) bacterial strains [1]. Complex interactions of *M. tuberculosis* with environmental and host genetic factors play a critical role in TB development. Host genetic factors explain, at least in part, why some people are more or less susceptible to infection [2]. Mannose-binding lectin (MBL) is an acute-phase serum protein in the collection family that recognizes a pathogen by its carbohydrate recognition domain. It is a key molecule of the innate immune system [3, 4]. MBL is encoded by MBL2, located on chromosome 10, and six MBL2 single nucleotide polymorphisms (SNPs) are associated with serum levels and/or functions of MBL. Three nonsynonymous nucleotide substitutions in exon 1 change the wild A allele to the three variant alleles (A/B, A/C, and A/D), which disrupt the collagenous structure and the formation of functional oligomers. The other alleles, H/L, X/Y, and P/Q, are distinguished by the SNPs in the promoter and 5’-untranslated regions, and the X allele shows the lowest transcriptional activity among them [5].

The impact of MBL gene polymorphism and the susceptibility to TB have been reported before, though the findings are inconsistent between studies. Polymerase chain reaction with sequence-specific primer (PCR-SSP) was often used in these studies. Some studies have found that low levels of serum MBL could reduce tubercle bacilli infections [6, 7], while another study has suggested the opposite [8, 9]. The results of association studies and the risk of TB are also inconsistent among studies.

We explored whether MBL2 polymorphisms or MBL levels are associated with the development of pulmonary TB (PTB) in China, a country with high TB prevalence. In this study, MBL2 -221X/Y and exon l codon 54 A/B gene polymorphism and the susceptibility to TB were evaluated by P**C**R-RFLP technology. At the same time, MBL levels were also detected.

## Methods

### Study population

From January 2010 to December 2011, a total of 112 inpatients (aged 19 to 91 years) diagnosed with PTB consisting of 78 (69.6%) men and 34 (30.4%) women were enrolled in this study. Additionally, 120 healthy donors who had undergone a physical examination in the hospital and were designated healthy were enrolled in this study. The cases and the controls were selected by simple random sampling technique according to random number table method. The inclusion criteria for the pulmonary TB cases was as follows: (1) the diagnosis of TB was based on clinical and radiological findings together with the positive identification of acid-fast bacilli (AFB) or culture; (2) the patient was registered from January 2010 to December 2011; (3) the patient was willing to participate in the study; and (4) complete medical records were available; The exclusion criterion for the cases and controls was as follows: people with comorbidities such as lung carcinoma, asthma, diabetes mellitus and other immunosuppressive conditions or people without providing correct information.

Clinical features of pulmonary TB including hemoptysis, fever, a history of treatment with anti-TB drugs and cavitary lesion as well as age, gender were reviewed.

### Estimation of sample size

Sample size estimation was based on an estimated XA frequency of 12%, OR = 2.7, α = 0.05 (paired) and β = 0.20. Based on the above assumptions, 112 patients and 120 healthy controls were enrolled.

### Sample collection and DNA extraction

Peripheral blood, 4 ml, was obtained from each patient and control. Samples were centrifuged for 10 min at 3000 g to separate serum. Ethylene diamine tetraacetic acid (EDTA) anticoagulant tubes were used to collect another 2 ml venous blood samples from each participant. A blood DNA isolation kit was used to extract the peripheral white blood cell genome (MBI, USA). The subsequent DNA extraction procedures were performed according to the manufacturer instructions and DNA were stored in a refrigerator at -20 °C.

### MBL2 -221X/Y and exon l codon 54 A/B genotyping

We identified the X/Y and A/B gene by PCR-RFLP using the primer 5'-ACCTGGGTTTCCACTC ATTCTCAT-3' and 5'-CCCCAGGCAGTTTCCTCTGGAAGG-3'. The procedure for PCR was 4 min at 95 °C, followed by 35 cycles (95 °C for 30 s, 63 °C for 60 s, 72 °C for 30 s) and 72 °C extension for 5 min. The enzyme digestion reaction system had a volume of 20 μL, including 5 μL purified PCR product, 2 μL 10X buffer, 12 μL ddH_2_O,1.0 μL of the corresponding restriction endonuclease, Btg I (NEB, USA) to genotype X/Y and BshN I (MBI, USA) to genotype A/B. The digestion reaction system was kept at 37 °C overnight. Five μL of enzyme-digested product was applied to 2% agarose gel. Gel imaging processing system was used to observe the electrophoresis results of the digested PCR products to determine the genotype.

### Serum MBL level assay

Serum MBL levels in samples from 112 patients and 120 healthy controls were assayed by human MBL ELISA kit (R&D Systems, USA). Procedures were performed according to manufacturer’s instructions and serum MBL levels between different diplotype groups were compared.

### Statistical analysis

SPSS for Windows, Version 17.0 (Statistical Package for Social Sciences, Chicago, IL, USA) was used for statistical analyses. The chi-square test was used to analyze the data of the genotype frequencies among the different groups. The risk of disease associated with MBL2 genotype was estimated using the calculation of odds ratios with 95% of confidence interval(CI). Serum MBL levels in the TB/healthy control group were analyzed by *t* test. One-way analysis of variance (ANOVA) was used to compare the MBL levels among the MBL2 diplotype-based groups. The association between the MBL2 polymorphisms and serum MBL levels was analyzed using a multiple regression model. A *P* value of < 0.05 was considered statistically significant.

## Results

### Distribution of MBL2 -221X/Y and exon l codon 54 A/B haplotypes in the Chinese population

The study participants included 112 PTB patients and 120 healthy controls. The TB patient group and the healthy control group exhibited no statistically significant differences (*P* > 0.05) in age, educational background, alcohol consumption or smoking (data not shown). The YA, XA, and YB haplotypes were observed in the Chinese population (Table 1). The TB patients were divided into different groups according to the demographic characteristics. The resistant genotypes were more frequently found in the younger patients and retreatment cases with TB in MBL gene sites −221 (OR value: 0.4 and 2.78 respectively) or codons 54 site (OR value: 0.31 and 2.71, respectively). No statistically significant differences (*P* > 0.05) were found among the different TB groups with respect to gender, or occurrence of fever, cavity, and hemoptysis (Table 2).

**Table 1:** Haplotypes and their frequencies in the healthy control (*n* = 120) and TB patients (*n* = 112)

Haplotype	TB patients		Healthy control	
	n	Frequency	n	Frequency
YA	132	0.59	170	0.71
YB	35	0.16	26	0.11
XA	57	0.25	44	0.18

**Table 2:** MBL2 gene polymorphisms in TB patients with different demographic characteristic (*n* = 112)

Characteristic	Total	−221		*P* value	exon 1 (codons 54)		*P* value
		Y/Y	Y/X+ X/X	OR (95%CI)	A/A	A/B + B/B	OR (95%CI)
Gender							
Male	78	45	33	0.687	59	19	0.135
Female	34	21	13	0.84 (0.37–1.92)	21	13	1.92 (0.81–4.56)
Age							
≤ 45	72	37	35	**0.03**	46	26	**0.018**
> 45	40	29	11	**0.40 (0.17–0.92)**	34	6	**0.31 (0.11–0.84)**
Initial TB							
Yes	65	45	20	**0.009**	52	13	**0.018**
No	47	21	26	**2.78 (1.27–6.07)**	28	19	**2.71 (1.17–6.30)**
Fever							
Yes	33	20	13	0.816	25	8	0.512
No	79	46	33	1.10 (0.48–2.53)	55	24	1.36 (0.54–3.45)
Cavity							
Yes	45	27	18	0.850	33	12	0.715
No	67	39	28	1.08 (0.50–2.32)	47	20	1.17 (0.50–2.72)
Hemoptysis							
Yes	46	24	22	0.225	35	11	0.362
No	66	42	24	0.62 (0.29–1.34)	45	21	1.48 (0.63–3.48)

*TB* tuberculosis, *OR* odds ratio, *CI* confidence interval, *P* values and ORs with confidence intervals (CIs) shown in bold type are statistically significant

Diplotypes YA/YA of MBL gene were more prevalent in the healthy control group than in the TB patient (*P* =0.038, OR, 0.57; 95%CI, 0.34-0.97), while diplotypes XA/XA were less frequently observed in the healthy control group (*P* =0.007, OR, 6.42; 95%CI, 1.39-29.67). The results are shown in Table 3.

**Table 3:** MBL2 diplotypes in controls and patients with PTB

Diplotypes	Patients (*n* = 112)		Controls (*n* = 120)		P	OR (95%CI)
	No.	(%)	No.	(%)		
YA/YA	40	(35.7)	59	(49.2)	**0.038** ^**a**^	**0.57 (0.34–0.97)**
YA/XA	29	(25.9)	35	(29.1)	0.577	0.85 (0.47–1.51)
YB/YB	3	(2.7)	2	(1.7)	0.674^b^	1.62 (0.27–1.90)
YA/YB	23	(20.5)	17	(14.1)	0.199	1.56 (0.78–3.11)
XA/XA	11	(9.8)	2	(1.7)	**0.007** ^**c**^	**6.42 (1.39–29.67)**
XA/YB	6	(5.4)	5	(4.2)	0.670	1.30 (0.38–4.39)

^a^ TB development associated with YA/YA was assessed by odds ratios (OR) with non-YA/YA as a reference

^b^Calculated by Fisher’s exact test

^c^TB development associated with XA/XA was assessed by odds ratios (OR) with non-XA/XA as a reference. *P* values and ORs with confidence intervals (CIs) shown in bold type are statistically significant

### MBL levels in TB patients and control

Serum MBL levels were range from 162.4 to 4963.1 ng/mL. The average of serum MBL levels were significantly higher in patients with TB compared to control subjects (*P* < 0.01), 2309.1 and 1721.1 ng/mL, respectively.

In this study we used ≤500 ng/mL as the cut-off for low or partial MBL deficiency. The prevalence of MBL deficiency in TB patients and healthy control was 8%, 27.5%, respectively.

### Association of MBL2 haplotypes with serum MBL levels in the population

**Fig. 1:**
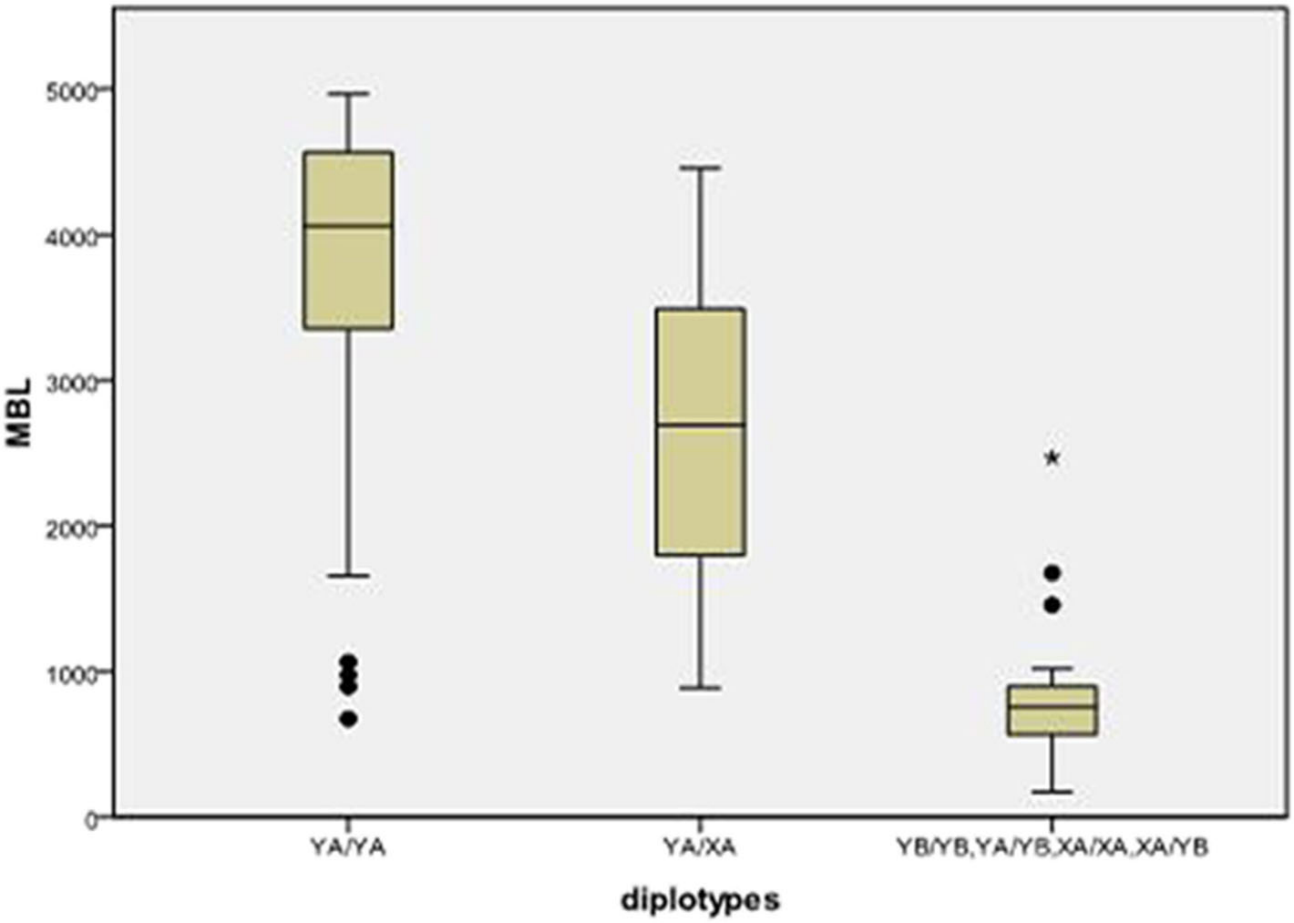
Serum MBL levels and diplotypes in patients with TB. MBL levels were different among the three groups (*P* < 0.01), and the difference was significant between any two of the three group

**Fig. 2:**
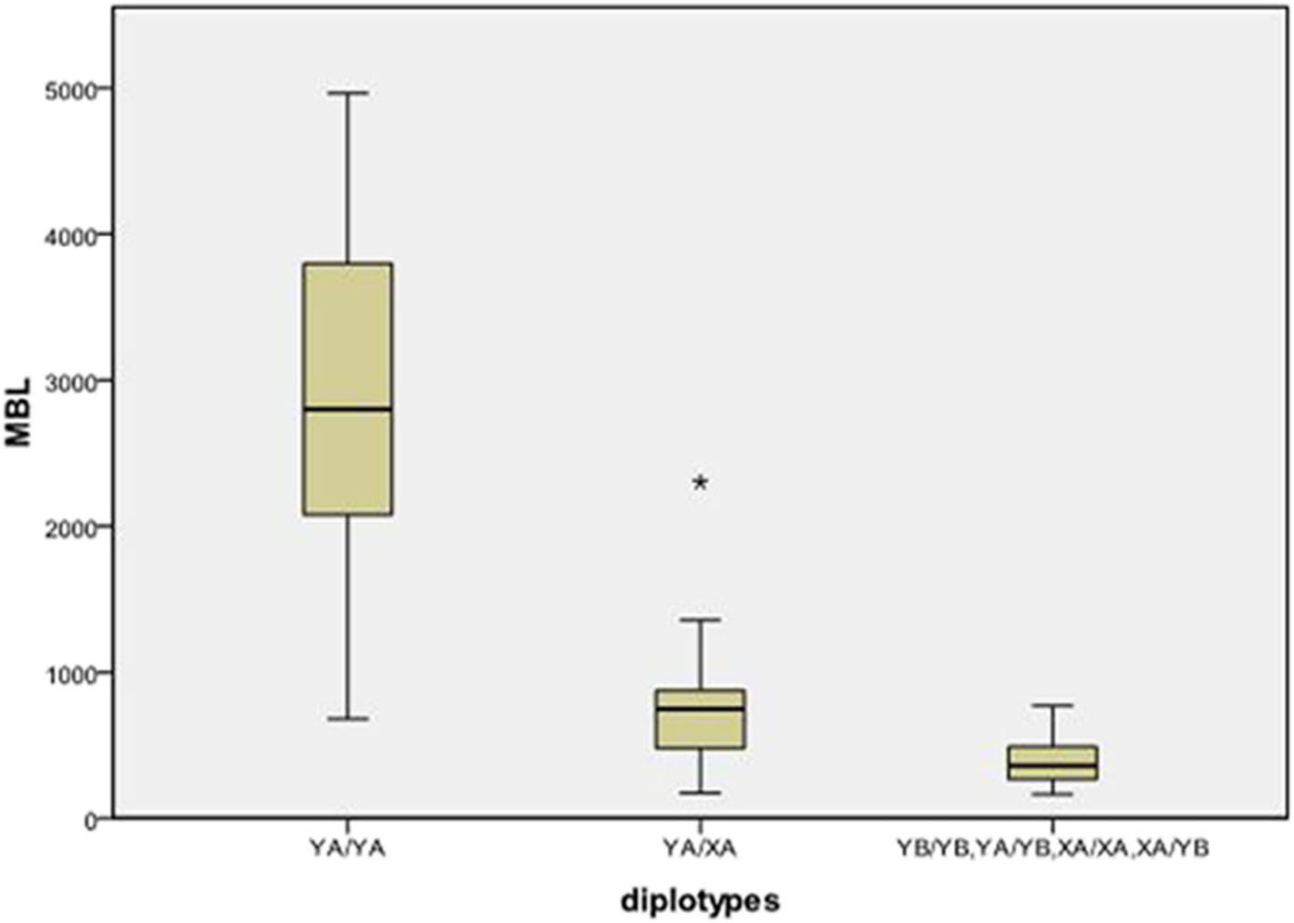
Serum MBL levels and diplotypes in healthy control. MBL levels were different among the three groups (*P* < 0.01), and the difference was significant between any two of the three groups. 93.2% of the YA/YA carrying individuals and ≤500 ng/mL in 84.6% of the XA/XA- or B- carrying individuals in healthy controls

The distribution of MBL serum levels closely correlated with the different MBL2 diplotypes in the healthy control group or in the TB patient (Figs. 1 and 2) (*P* < 0.01). According to MBL levels, The MBL2 diplotypes were divided into three groups, YA/YA, XA/YA, XA/XA or B- carrying diplotypes. We observed MBL levels were significantly different between any two of the three groups (*P* < 0.01). A marked decrease of the serum MBL levels was associated with the presence of XA/XA or B- carrying individuals. MBL levels were ≤500 ng/mL in 84.6% (22/26) of the XA/XA- or B- carrying individuals in healthy controls. So based on our results, XA/XA or B- carrying diplotypes were considered low or deficient genotypes.

## Discussion

Because of strong linkage disequilibrium, seven haplotypes are commonly observed, and often classified into three groups of higher-producing (HYPA, LYPA, and LYQA), lower-producing (LXPA), and non-functional (LYPB, LYQC, and HYPD) haplotypes [10]. In this study, MBL gene polymorphisms were evaluated (variants in -221Y/X and exon l codons 54 A/B). The YA, XA and YB haplotypes were observed in the Chinese population. Genotypes YA/YA of MBL gene were more prevalent in the healthy control group than in the TB patients (*P* <0.05), while genotypes XA/XA was six times more frequent in patients with TB than in the healthy control group (*P* <0.01). The YA/YA may be a protected diplotype against TB compared to XA/XA which may be a susceptible diplotype to TB. This is consistent with the findings of Minako Hijikata and Rosanna Capparelli study [10, 11]. But another study has suggested that X,Y alleles were not associated with PTB from a population in China [12]. In view of the evolutionary history of *M. tuberculosis*-human interactions, many of the genetic variants occur at relatively high frequencies, but negative consequences to the host could limit selective sweeps. Some researchers highlight cases where evidence of selection pressure is established as a sign that the genetic variant indeed has a strong phenotypic influence on TB [12]. According to different demographic characteristics in TB patients, our study revealed that the promoter -221(Y/X) and exon l codons 54 (A/B) mutation of the MBL gene were significantly different between different ages, and initial or retreatment TB cases. Meanwhile, there was no significant difference among different gender, the presence of fever, cavity, or hemoptysis. The resistant diplotype was more frequently found in the younger patients and retreatment cases with TB in MBL gene sites -221Y/X or codons 54 A/B. Our study revealed that the polymorphisms of MBL gene may be associated with recurrence of TB, and increases the chances of TB infection in the younger age group. This result is a little different from another study conducted in China [13]. All these findings suggest that the occurrence of TB infection is a complicated process.

MBL is a calcium-dependent lectin which is secreted by the liver and binds to several sugars (mannose and N-acetyl-D-glucosamine, in particular) expressed on a wide range of pathogens. MBL has been shown to play an important role in host defense against pathogens including bacteria, fungi, parasites and viruses [14–17]. A study showed that the reduction in serum MBL could increase the chances of TB infection [10]. But it has been proposed in another study that high MBL serum levels may lead to increased TB infections by promoting *M. tuberculosis* opsonization. By binding to *M. tuberculosis*, MBL acts as an opsonin, enhances both complement dependent and independent phagocytosis, and promotes inflammation resulting in the release of cytokines. This has been strengthened by studies demonstrating that MBL enhances phagocytic activity against other mycobacteria and demonstration of a protective effect of MBL deficiency against at least some forms of *Mycobacterium leprae* infection [18]. MBL genotype may be associated with the serum MBL level in the population. Promoter-221(Y/X) mutation can lower the serum MBL level and subsequently increase susceptibility to TB. In this study, Serum MBL levels were significantly lower in control than in patients with TB. MBL deficiency appeared to protect against tuberculosis. We used ≤500 ng/mL as the cut-off for low or partial MBL deficiency. XA/XA or B- carrying diplotypes were considered low or deficient genotypes. Our classification almost matched with the definition by Albert RK [19].

A literature review and meta-analysis [20] of 17 clinical trials considering the effect of MBL2 genotype and/or MBL levels and TB infection, reported no significant association between MBL2 genotype and PTB infection. Serum MBL levels were shown to be consistently elevated in the setting of TB infection. This may indicate that high MBL levels may be associated significantly with susceptibility to PTB infection. A recent study showed that passive smoking, cooking with solid fuel, and polymorphisms of MBL gene were associated with susceptibility to TB in non-smokers [21]. The outcome of TB infection and disease depends on interactions between host and pathogen genotypes. Further studies are needed to explore details of the mechanisms of association.

There are six known polymorphism within MBL2 such as Y/X, H/L, P/Q, B, C, D. The B allele is common in Asian population [22]. No or less mutation was found in C, D allele according to our preliminary experiment. At present, only Y/X and A/B polymorphisms were included in this study. We did not analyze the impact of other gene. There may be gene-gene interactions among them associated with TB susceptibility. More gene polymorphism sites and expanded sample size will be analyzed in the future. Although there are some shortages to this study, the possible impacts of non-genetic factors such as age, educational background, alcohol consumption or smoking were adjusted. So the results shown in this study should be reliable.

## Conclusion

Our study revealed that the promoter -221 (Y/X) and exon l codons 54 (A/B) mutation of the MBL gene was associated with susceptibility and recurrence of TB. We found that YA/YA, associated with high plasma MBL levels, protected against the development of new cases of PTB. Although many factors are involved in the occurrence and spread of TB such as environment, heredity, and environment within the host, further in-depth understanding of the generation mechanism of TB may provide better ideas to control TB.

## Abbreviations

MBL: Mannose-binding lectin
MDR: Multidrug-resistant
P**C**R-RFLP: Reaction–restriction fragment length polymorphism
PTB: Pulmonary TB
SNPs: Single nucleotide polymorphisms
XDR: Extensively drug-resistant
